# A Novel Camera Calibration Method Based on Polar Coordinate

**DOI:** 10.1371/journal.pone.0165487

**Published:** 2016-10-31

**Authors:** Shaoyan Gai, Feipeng Da, Xu Fang

**Affiliations:** 1 Key Laboratory of Measurement and Control of Complex Systems of Engineering, Ministry of Education, School of Automation, Southeast University, Nanjing 210096, China; 2 State Key Laboratory of Digital Manufacturing Equipment & Technology, Huazhong University of Science & Technology, Wuhan 430074, China; 3 Key Laboratory of Modern Agricultural Equipment and Technology(Jiangsu University), Ministry of Education, Zhenjiang 212013, China; West Virginia University, UNITED STATES

## Abstract

A novel calibration method based on polar coordinate is proposed. The world coordinates are expressed in the form of polar coordinates, which are converted to world coordinates in the calibration process. In the beginning, the calibration points are obtained in polar coordinates. By transformation between polar coordinates and rectangular coordinates, the points turn into form of rectangular coordinates. Then, the points are matched with the corresponding image coordinates. At last, the parameters are obtained by objective function optimization. By the proposed method, the relationships between objects and cameras are expressed in polar coordinates easily. It is suitable for multi-camera calibration. Cameras can be calibrated with fewer points. The calibration images can be positioned according to the location of cameras. The experiment results demonstrate that the proposed method is an efficient calibration method. By the method, cameras are calibrated conveniently with high accuracy.

## Introduction

With the development of photo-electronics, image processing, information sensing, signal processing, and electronics, digital camera is becoming increasingly relevant in science and technology [1–14]. Camera calibration is an essential step in computer vision, image processing, and optical measurement [1–14], which makes it possible to obtain metric information of the object from the projections on the image plane. The accuracy of vision system is very sensitive to the camera parameters [15–18]. A tiny error in estimating camera parameters may adversely affect the whole system performance.

Camera calibration has been widely studied, which falls into several categories [18–19]. One category of the methods is called coplanar approaches. These methods rely on calibration points, which are on a planar template in a single depth. These approaches are either computationally complex or fail to provide result solutions for one camera parameters or more, e.g., the image center, the scale factor, or lens distortion coefficients[19]. The category of calibration methods based on World-reference are classical approaches, which requires a set of calibration points with two dimensional image coordinates and corresponding of three dimensional world coordinates [20–22]. Zhang [23] proposed a flexible calibration method, which only requires a few images of a two-dimensional calibration plate taken from different orientations. Based on this method, a set of optimal conditions is proposed to improve the calibration results accurately[24]. The disadvantage of these methods is that a complex and high-precise calibration template is used to achieve precise 3D measurements. One-dimension calibration method is also studied [25]. By which, a 1D calibration object is placed at several positions and in different orientations[25].

Many researchers focus on the camera modeling and analysis, which is very important for stereo vision and display[26–31]. Yang and Song model two kinds of cameras, a parallel and a converged one, and analyze the difference between them in horizontal and vertical parallax[26]. The conclusion is that converged arrays are more suitable for short-distance. The work can be guidance to camera arrays applications. And it is pointed that, for future work on the camera array, it is necessary to focus on camera calibration and visual stereo-video evaluation. In reference[27], two types of stereo cameras are studied, which are parallel and toed-in cameras. The objective shooting quality evaluation criteria over short distance is proposed. Furthermore, three shooting conditions (macro shooting, short, and long distance shooting) are discussed in reference [28]. The shooting quality of stereo cameras can be evaluated effectively by the proposed approach. In reference [29], a full-reference metric for quality assessment of stereoscopic images is proposed. It is based on the binocular difference channel and binocular summation channel.

Reference [31] use point correspondences between model plane and image to calculate the homograph and distortion coefficients. A calibration process is applied, which is non-iterative with no risk of local minima. It’s a one-shot algorithm that can be solved by linear least-squares technique.

Generally, the 3D world coordinates and 2D image plan coordinates are related by calibration objects. They are in Cartesian coordinates usually. But some images are very complex, and cannot be represent in rectangular coordinate system. For example, rose line, Archimedes line, etc. For these images, the method based on rectangular coordinates cannot be used generally. But the characteristics of the curve is obvious. They are suitable for the feature point extraction and matching. Thus in this paper, polar coordinates are introduced to indicate the location of the calibration points. The coordinates of the points are obtained in polar coordinates, and then converted into rectangular coordinate system. The point can be easily expressed. The calibration can be applied without increasing system complex.

Polar coordinate images are suitable for multiple-camera calibration. The calibration object for multiple-camera has always been a problem: the 3D calibration objects may be hidden partly in one of the camera images; Two-dimension calibration plates are designed large and complex due to the scope of cameras in multiple locations. Two-dimensional flexible spliced calibration board are studied [32, 33]. Also one-dimensional calibration objects are studied [34, 35, 36] to solve the problems However there are movement restrictions in these solutions. Theodolite is used to obtain the precise and relative position [37]. However, additional equipment and complex operations is required.

In this paper, a whole solution of calibration by polar coordinates is presented. This method is in a novel view of calibration, that is the polar coordinates. The relationship in polar coordinates image is relatively simple, which can be easily used in the multiple-camera calibration plate. Furthermore, the layout of the polar coordinates calibration plate can be designed according to the camera position. Thus the manufacture difficulty of calibration objects is reduced. And the complexity of the calibration is simplified.

The method is useful in practice. Unlike the traditional methods based on rectangular coordinate system, the calibration board is designed in polar coordinates, and the difficulties in multiple-camera calibration is overcome. In the second section, the camera model and the calibration method is proposed in detail. In the third section, the simulation experiment and real experiment are presented. In the last section, the method of this paper is summarized.

## Methods

### Pinhole projection model

A camera is modeled by the usual pinhole model, in which case the relationship between a 3D point (*X*_w_,*Y*_w_,*Z*_w_) and its image projection (*u*,*v*) is given by

$$\rho \left(\begin{array}{c} u\\ v\\ 1 \end{array}\right)=\left(\begin{array}{cccc} f_{x} & -f_{\text{x}}\;\cot\theta & u_{0} & 0\\ 0 & f_{\text{y}}\;\sin^{-1}\theta & v_{0} & 0\\ 0 & 0 & 1 & 0 \end{array}\right)\left(\begin{array}{cc} R & T\\ 0^{T} & 1 \end{array}\right)\left(\begin{array}{c} X_{\text{w}}\\ Y_{\text{w}}\\ Z_{\text{w}}\\ 1 \end{array}\right)=\left(\begin{array}{cc} M_{1} & 0 \end{array}\right)\left(\begin{array}{cc} R & T\\ 0 & 1 \end{array}\right)\left(\begin{array}{c} X_{\text{w}}\\ Y_{\text{w}}\\ Z_{\text{w}}\\ 1 \end{array}\right)=\left(\begin{array}{cc} M_{1} & 0 \end{array}\right)M_{2}\left(\begin{array}{c} X_{\text{w}}\\ Y_{\text{w}}\\ Z_{\text{w}}\\ 1 \end{array}\right)=H\left(\begin{array}{c} X_{\text{w}}\\ Y_{\text{w}}\\ Z_{\text{w}}\\ 1 \end{array}\right)$$ (1)

where

$$M_{1}=\left(\begin{array}{ccc} f_{\text{x}} & -f_{\text{x}}\;\cot\theta & u_{0}\\ 0 & f_{\text{y}}\;\sin^{-1}\theta & v_{0}\\ 0 & 0 & 1 \end{array}\right),M_{2}=\left(\begin{array}{cc} R & T\\ 0^{T} & 1 \end{array}\right),H=\left(\begin{array}{cc} M_{1} & 0 \end{array}\right)M_{2}$$

where (*X*_w_,*Y*_w_,*Z*_w_) is the 3D coordinates of the point at the world coordinate system. In the camera image plan, the point image is produced at point (*u*,*v*). *ρ* is an arbitrary scale factor. *f*_x,_ *f*_y,_ *u*_0,_ *v*_0_, *θ* are camera intrinsics, which is included in the camera intrinsic matrix *M*_1_, where *f*_x,_ *f*_y_ are the scale factors in image abscissa and vertical axes respectively, and *u*_0,_ *v*_0_ define the coordinates of the principal point in the image plan of the camera, and *θ* is the parameter describing the skewness of the two image axes. *R*, *T* are the rotation and translation matrixes, which comprise the camera extrinsic matrix *M*_2_, which define the spatial relationship of camera and the world coordinate system. *R* is a general 3×3 orthogonal rotation matrix, and *T* is a 3×1 translation matrix. *M*_1_ and *M*_2_ are camera intrinsic matrix and extrinsic matrix respectively. *H* is the perspective projection matrix (3×4), which is the product of *M*_1_ and *M*_2_.

### Calibration in polar coordinate

In view of expressing graphics in polar coordinates, there are two ideas:

1. The world coordinates and computer image coordinates are both expressed in polar coordinates;
2. The world coordinates are expressed in polar coordinates, which are converted to world coordinates in the calibration process. Computer image coordinates are in rectangular coordinates.

In the case of method 1, the system have to be modified to satisfy Eq (1). The left of Eq (1) should be in polar coordinates. However the image hardware implementation is a representation of pixel rows and columns, which is arranged according to the rectangular coordinate system. Thus we use the second method.

**Fig 1:**
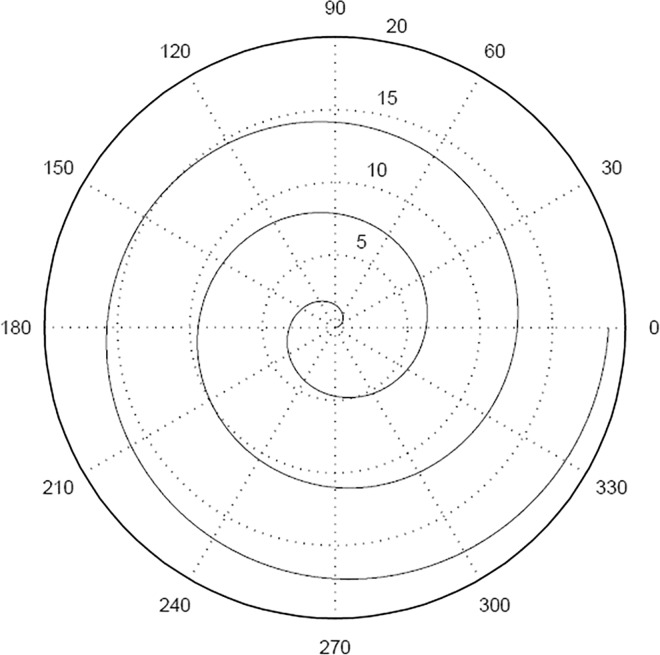
Archimedes curve.

**Fig 2:**
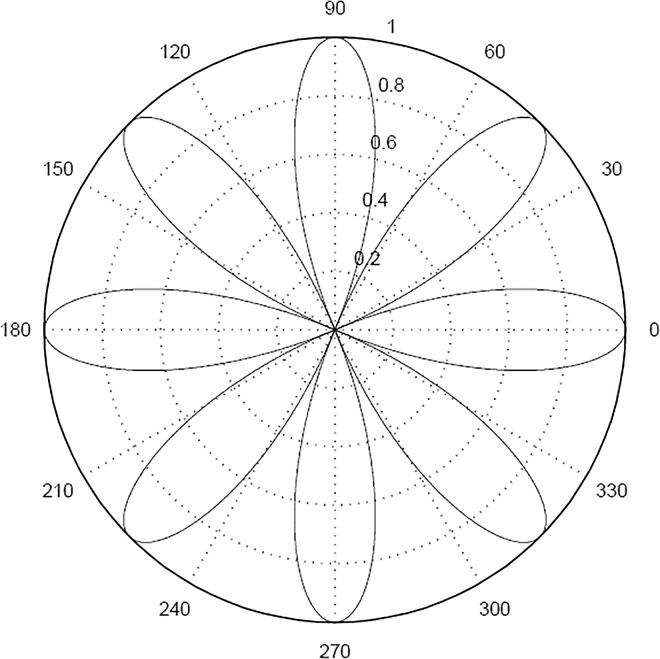
Rose Curve.

Some calibration image patterns, e.g. Archimedes line and rose line in Fig 1 and Fig 2, are suitable for polar coordinates. The characteristic point are in form of polar coordinates. Then the rectangular coordinates of the points are obtained by coordinate transformation. And the corresponding image coordinates are matched with the points. By this simple transformation, it will not increase the complexity of the system.

### Computation of H

As we known it in Eq (1), *H* is a 3×4 matrix. The calibration image base on polar coordinate is in a plane. Without loss of generality, the plane is *Z*_w_ = 0. Then Eq (1) becomes

$$\rho \left(\begin{array}{c} u\\ v\\ 1 \end{array}\right)=H\left(\begin{array}{c} X_{\text{w}}\\ Y_{\text{w}}\\ 0\\ 1 \end{array}\right)=\left(\begin{array}{ccc} h_{1} & h_{2} & h_{3} \end{array}\right)\left(\begin{array}{c} X_{\text{w}}\\ Y_{\text{w}}\\ 1 \end{array}\right)$$ (2)

As can be seen from Eq (2), the third column of *H* is omitted since *Z*_w_ = 0. Thus *H* turned into a 3×3 matrix, which contains 9 unknowns. Since there is a scaling factor *ρ*, there are only eight unknown parameters. By four (or more) groups of corresponding points (*u*,*v*)—(*X*_w_,*Y*_w_,*Z*_w_), the matrix *H* can be calculated.

### Computation of camera parameters

By Eq (1) and Eq (2), *H* can be expressed as a column-matrix form, as follows

$$\lambda \left(\begin{array}{ccc} h_{1} & h_{2} & h_{3} \end{array}\right)=\lambda H=M_{1}M_{2}=M_{1}\left(\begin{array}{ccc} r_{1} & r_{2} & T \end{array}\right)$$ (3)

Where *r*_1_, *r*_2_ is the first and second columns of orthogonal rotation matrix *R*. *T* is the translation matrix. By the nature of orthogonal matrix, we have

$$r_{1}{}^{\text{t}}r_{1}=1,\;r_{2}{}^{\text{t}}r_{2}=1\;\mathrm{and}\;r_{1}{}^{\text{t}}r_{2}=0$$ (4)

Where t denotes matrix transposition. From Eq (2) and Eq (3), we have

$$h_{1}{}^{\text{t}}M_{1}{}^{\text{-t}}M_{1}{}^{\text{-}1}h_{2}=0$$ (5)

$$h_{1}{}^{\text{t}}M_{1}{}^{\text{-t}}M_{1}{}^{\text{-}1}h_{1}=h_{2}{}^{\text{t}}M_{1}{}^{\text{-t}}M_{1}{}^{\text{-}1}h_{2}$$

According to Eq (5), two equations can be obtained from each image. There are five unknowns in the intrinsic matrix *M*_1_. If there are three images, the intrinsic matrix can be determined with Eq (5). If there are more than 3 images, an optimal solution can be worked out.

Then, the extrinsic matrix can be obtained by the result of *M*_1_ and Eq (4), as follows

$$\{\begin{array}{l} r_{1}=\lambda M_{1}{}^{\text{-}1}h_{1}\\ r_{2}=\lambda M_{1}{}^{\text{-}1}h_{2}\\ r_{3}=r_{1}\times r_{2}\\ T=\lambda M_{1}{}^{\text{-}1}h_{3} \end{array}$$ (6)

where *λ* = 1/‖*M*_1_^-1^*h*_1_‖ = 1/‖*M*_1_^-1^*h*_2_‖. The result is a primary solution, without lens distortion.

Furthermore, we consider lens distortion, which can be express as

$$\{\begin{array}{c} u_{\text{d}}=u+k_{1}u(u^{2}+v^{2})+k_{2}u{\left(u^{2}+v^{2}\right)}^{2}\\ v_{\text{d}}=v+k_{1}v(u^{2}+v^{2})+k_{2}v{\left(u^{2}+v^{2}\right)}^{2} \end{array}$$ (7)

Where (*u*,*v*) and (*u*_d_,*v*_d_) are the ideal pixel image coordinates and the real image coordinates, respectively. *k*_1_,*k*_2 _are coefficients of radial distortion.

Then, the optimal solution with radial distortion can be calculated. The objective function for optimization is defined as

$$\sum_{i=1}^{n}\sum_{j=1}^{m}\left\|p_{ij}-\hat{p}(M_{1},R_{i},T_{i},u_{\text{d}ij},v_{\text{d}ij},k_{1},k_{2})\right\|$$ (8)

where *p*_*ij*_ is the actual coordinates of the *j*-th point in *i*-th image, (*u*_d*ij*_,*v*_d*ij*_) is the pixel image coordinates, $\hat{p}(M_{1},R_{i},T_{i},u_{\text{d}ij},v_{\text{d}ij},k_{1},k_{2})$ is the calculation result of coordinates, *n* denotes the total number of images, and *m* denotes the number of points in each image.

### Multiple-camera calibration

**Fig 3:**
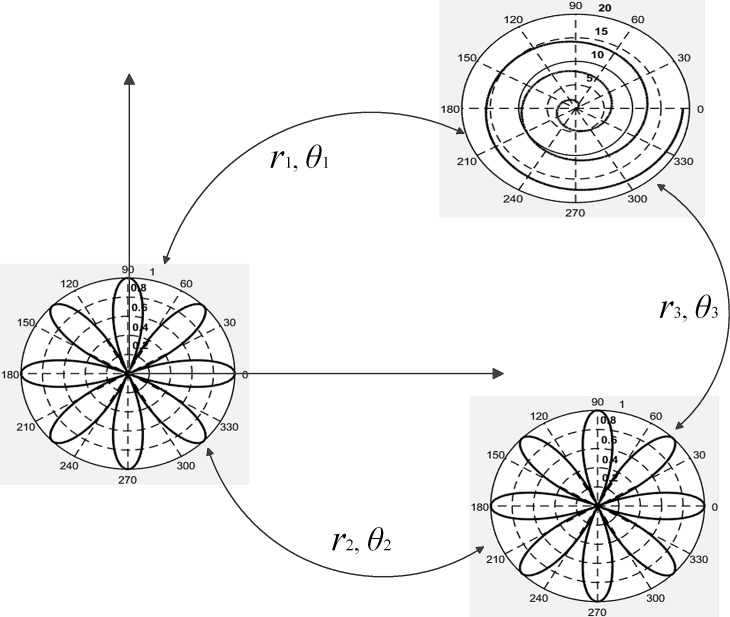
Multi-camera calibration board based on cameras’ positions.

Due to the simple transform relationship in polar coordinates, it is convenient for multiple-camera calibration. Calibration board can be designed as the graphics shown in Fig 3. By the relationship between each board image and its center (*r*_*i*_,*θ*_*i*_), it is easy to determine the absolute position of the points in polar coordinates.

The great advantage of polar coordinates in multiple-camera calibration is as follows.

**Fig 4:**
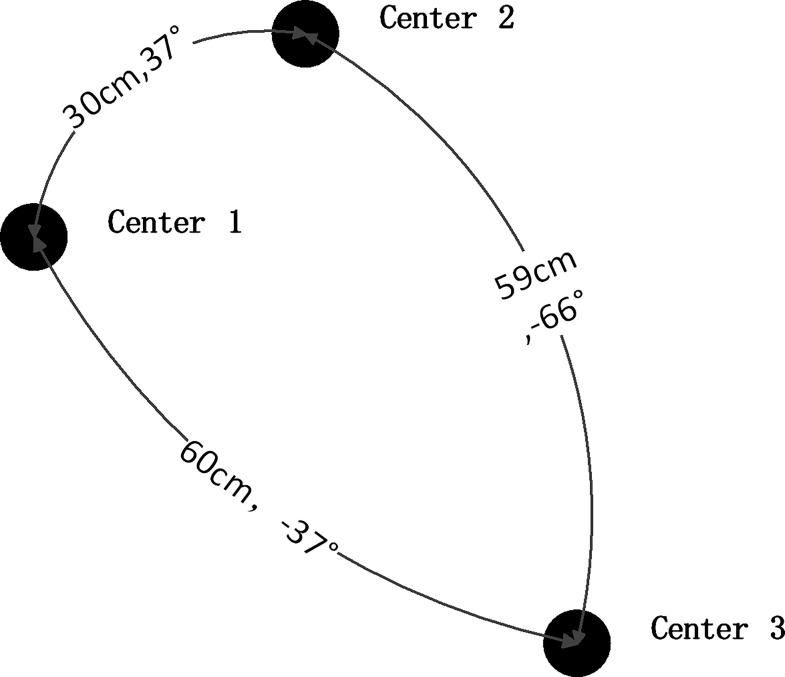
Multi-camera calibration board with known positions of centers.

It does not require common areas for all the cameras. For example, for camera 1, the center of the world coordinates is set to polar coordinates center 1. For camera 2, the center of the world coordinates is set to polar coordinates center 2. For camera 3, the center of the world coordinates is set to polar coordinates center 3. The center of the world coordinates of camera 2 and 3, is about the position of calibration board center. In this case, the calibration board can be changed according to the location of cameras. In other word, the location is not fixed in advance, but on or after the image is snapped. Thus it solves the problem of accumulated error caused by transfer matrix. As shown in Fig 4, center of polar coordinates 1 is (0, 0) in world coordinate, center of polar coordinates 2 is (24, 17) in world coordinate, and center of polar coordinates 3 is (48, -36) in world coordinate. Thus the world coordinate of the cameras is the same one. More than the traditional multiple camera calibration methods, where it requires public viewing area between two cameras [35,36], the proposed approach is free of public viewing area. It is not considered that data may be lost because cameras are of imperfect convergence configuration. The reason is that the calibration distance is shorter than the retrieval distance of the object. Thus there is no or few data-missing problem. The flexibility of calibration is enhanced to a large extent.

## Experiment Results

### Experiment 1 (Simulation)

In the following simulation experiments, the calibration objects is in form of Archimedes curve, as shown in Fig 1. The parameters are *p* = *θ* (*θ* = 0:6*π*), *θ* = 45° × *k*, *k* is integer, and *θ* ≥ 225°. A set of points, which are 12 points, are made as feature points.

The intrinsic matrix *M*_1_ is:

$$M_{1}=\left(\begin{array}{ccc} f_{\text{x}} & -f_{\text{x}}\;\cot\theta & u_{0}\\ 0 & f_{\text{y}}\;\sin^{-1}\theta & v_{0}\\ 0 & 0 & 1 \end{array}\right)=\left(\begin{array}{ccc} 1000 & 0 & 600\\ 0 & 1000 & 600\\ 0 & 0 & 1 \end{array}\right)$$

The extrinsic parameters of three images are:

1^st^ image:

        *α*_1_ = 16.9°, *β*_1_ = -11.6°, γ_1_ = -27.3°, *T*_1_ = [3,3,10]

2^nd^ image:

        *α*_1_ = 26.8°, *β*_1_ = -8.6°, γ_1_ = -38.5°, *T*_1_ = [2,2,10]

3^rd^ image:

        *α*_1_ = 28.3°, *β*_1_ = -19.9°, γ_1_ = 40.1°, *T*_1_ = [1,2,10]

**Fig 5:**
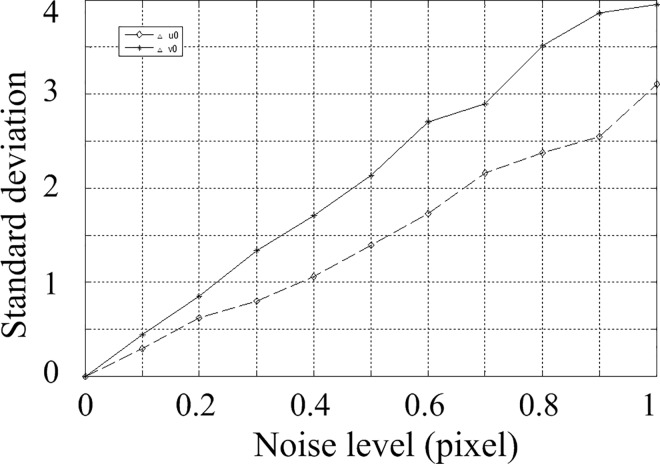
Standard deviations for parameters *u*_0_, *v*_0._

**Fig 6:**
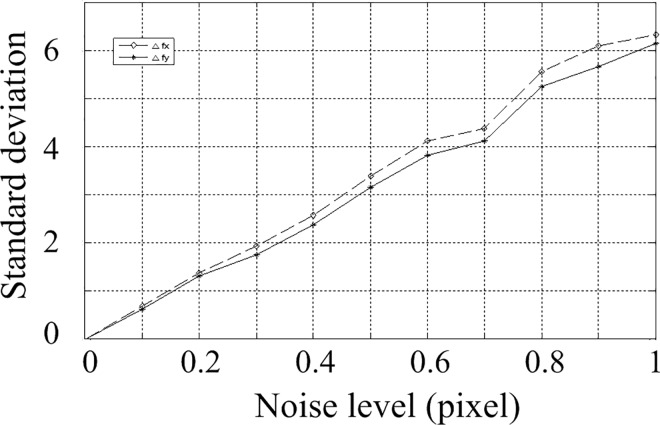
Standard deviations for parameters *f*_x,_ *f*_y._

**Fig 7:**
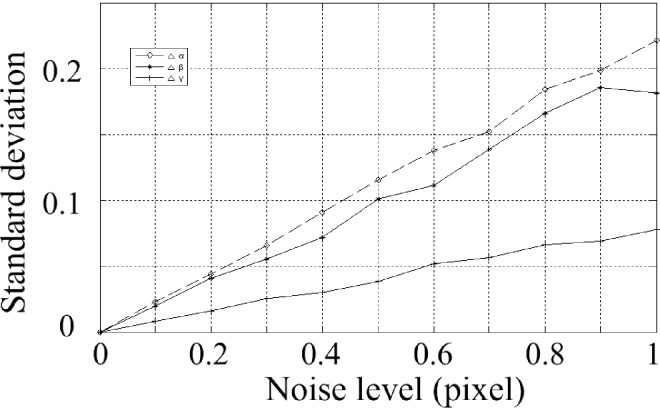
Standard deviations for parameters *α*,*β*,*γ*.

According to the above parameters, 3 calibration images are generated with a resolution of 2048 by 2048. In the projected image, gaussian noise with zero mean is added. The standard deviation of the noise is varying from 0 to 1 at an interval of 0.1 pixels. The calculation is performed 200 times under each noise level. The obtained parameters are shown. The Average of parameters under different noise levels are shown in Table 1 and Table 2, and the standard deviation is shown in Figs 5, 6 and 7. The horizontal ordinates of Figs 5–7 denote the noise level in pixel. The vertical ordinates denote the standard deviation of parameters. The standard deviation of *u*_0_, *v*_0_ is shown in Fig 5. The standard deviation of *f*_x,_ *f*_y_ is shown in Fig 6. The standard deviation of *α*,*β*,*γ* is shown in Fig 7.

**Table 1:** Average of intrinsic parameters under different noise levels.

NL	*f*_*x*_	*f*_*y*_	*u*_*0*_	*v*_*0*_
0	600.0000	600.0000	1000.0000	1000.0000
0.1	599.9834	599.9775	999.9802	1000.0040
0.2	599.8631	599.8718	999.9760	999.9273
0.3	600.0144	600.0189	1000.0778	1000.0089
0.4	599.8394	599.8087	999.8691	999.9723
0.5	599.7578	599.7790	1000.0098	999.8752
0.6	599.6746	599.6829	999.9355	999.8019
0.7	600.3498	600.1680	999.8610	1000.4103
0.8	599.8403	599.9039	1000.0818	999.8760
0.9	600.3305	600.3718	1000.1911	1000.0553
1.0	599.4579	599.5375	1000.0651	999.7686

**Table 2:** Average of extrinsic parameters under different noise level.

NL	*α*	*β*	γ	*T*_1_
0	16.9034	-11.5991	-27.2548	[3.0000 3.0000 10.000]
0.1	16.9031	-11.5981	-27.2550	[3.0004 2.9999 9.9996]
0.2	16.8989	-11.5978	-27.2540	[3.0003 3.0012 9.9980]
0.3	16.9043	-11.6056	-27.2539	[2.9986 2.9999 10.001]
0.4	16.8966	-11.5940	-27.2558	[3.0021 3.0005 9.9974]
0.5	16.9017	-11.6034	-27.2499	[2.9996 3.0020 9.9966]
0.6	16.8888	-11.5962	-27.2526	[3.0008 3.0031 9.9941]
0.7	16.9100	-11.6037	-27.2639	[3.0022 2.9935 10.003]
0.8	16.9039	-11.5907	-27.2534	[2.9988 3.0019 9.9979]
0.9	16.9109	-11.6122	-27.2544	[2.9964 2.9991 10.007]
1.0	16.9005	-11.5984	-27.2509	[2.9984 3.0035 9.9926]

It can be clearly seen that, intrinsic parameters deviation is of the order of magnitude of 0.01 to 0.1, while extrinsic parameters deviation is of the order of magnitude of 1. Since the value of intrinsic parameters is large, the proportion of the deviation may be small. For example, *f*_x_ *=* 1000, and the max deviation of *f*_x_ is 0.6, thus the proportion of the deviation is 0.6%. In case of the extrinsic parameters, the max deviation of *α* is 0.225, and the proportion of the deviation is 1.3%. In conclusion, the noise impact on the results is relatively small. The algorithm is of high stability.

**Fig 8:**
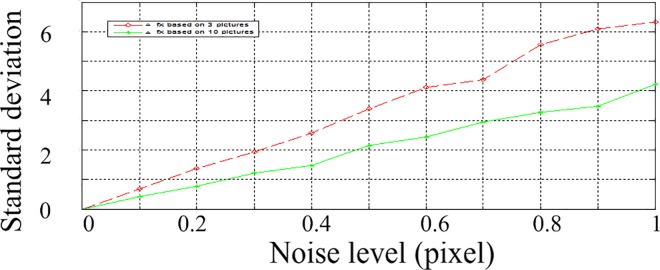
the standard deviations of *f*_x_ with 3 and 10 pictures.

**Fig 9:**
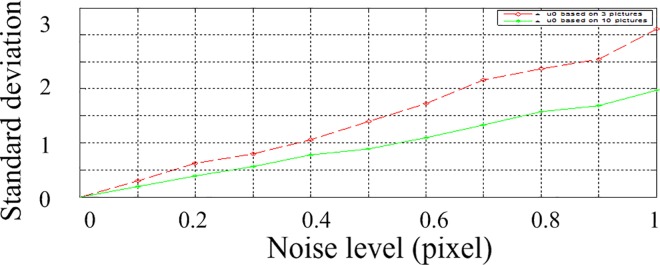
the standard deviations of *u*_0_ with 3 and 10 pictures.

**Fig 10:**
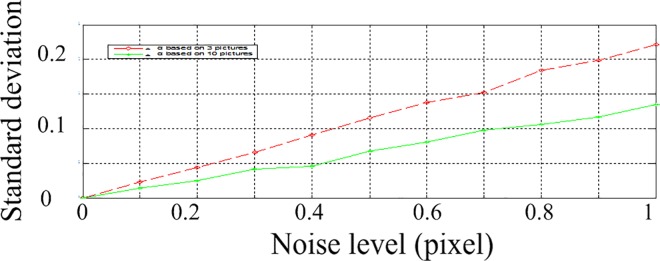
the standard deviations of *α* with 3 and 10 pictures.

To study the inference of the numbers of pictures, we try different numbers of pictures. The results of 3 pictures and 10 pictures are shown in Figs 8–10. The horizontal ordinates of Figs 8–10 denote the noise level in pixel. The vertical ordinates denote the standard deviation of parameters. The Standard deviations for intrinsic parameters *f*_x_, *u*_0_ and extrinsic parameters *α* are shown in Fig 8, Fig 9 and Fig 10, respectively.

As shown in Figs 8–10, the stability of 10 pictures is better than results of 3 pictures. But with 10 pictures, the calibration processing is complex significantly, while the improvement is not notable. In practice, 3–5 pictures are suitable for calibration applications.

### Experiment 2 (Real data experiments)

The real data experiments were carried out in our laboratory. To demonstrate the efficiency of the proposed method, camera calibration based on proposed calibration image and accurate calibration plate are conducted. And zhang’smethod, which is the classical calibration method, is also peformed.

**Fig 11:**
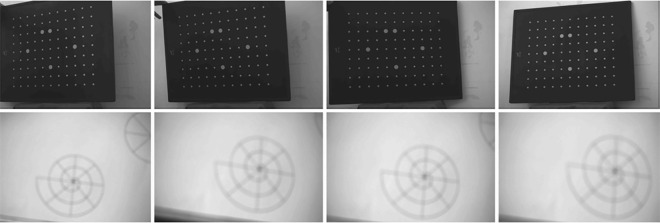
Real Pictures—the accurate calibration board is at the top, the polar coordinates calibration board is at the bottom.

A UNIQ UP-1800 camera with fixed focal length is placed in front of the calibration boards. The image resolution is 1380×1030 pixels. The training data for Zhang’s calibration is obtained from the accurate calibration board. While the training data for the proposed method is obtained from the polar coordinates calibration board. The images of calibration boards are shown in Fig 11, where the accurate board is at the top, and the polar coordinates board is at the bottom.

The results are listed as follows

The calibration results of intrinsic parameters by the proposed method:

$$M_{1}=\left(\begin{array}{ccc} 2720.9208 & 0 & 686.4637\\ 0 & 2725.6433 & 558.8436\\ 0 & 0 & 1 \end{array}\right)$$

The calibration results of intrinsic parameters by accurate calibration plate:

$$M_{2}=\left(\begin{array}{ccc} 2496.5098 & 0 & 683.5691\\ 0 & 2491.5819 & 523.8588\\ 0 & 0 & 1 \end{array}\right)$$

Both of algorithms obtain accurate calibration results. From a practical point of view, the proposed method is more simple and convenient, and the broad is of high applicability.

**Fig 12:**
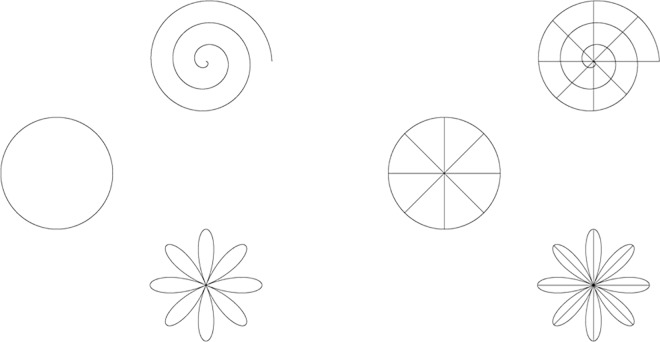
Multiple-camera calibration board.

The relationship between Polar coordinates images is simple. Thus Polar coordinates calibration images can be easily used for multiple camera calibration. The calibration can be performed by pre-made calibration board, as shown in Fig 12, the respective relationship of which is known before snapping images. Also the relationship can be calculated according to the snapped images, as shown in Fig 3. In this case, the calibration images can be positioned according to the location of the cameras.

The experiment results of multiple camera calibration are shown as follows.

The optimized parameters of left camera are

$$K=\left(\begin{array}{ccc} 2720.9208 & 0 & 686.4637\\ 0 & 2725.6433 & 558.8436\\ 0 & 0 & 1 \end{array}\right)$$

$$\left[R\;T\right]=\left(\begin{array}{cccc} 0.9540 & 0.0657 & 0.2926 & 11.67\\ 0.1263 & -0.9729 & -0.1936 & -8.62\\ 0.2720 & 0.2216 & -0.9364 & 41.63 \end{array}\right)$$

The optimized parameters of right camera are

$$K=\left(\begin{array}{ccc} 2679.2684 & 0 & 689.5\\ 0 & 2718.2683 & 514.5\\ 0 & 0 & 1 \end{array}\right)$$

$$\left[R\;T\right]=\left(\begin{array}{cccc} -0.9717 & 0.0622 & 0.2279 & -0.33\\ -0.0208 & -0.9835 & 0.1800 & -0.47\\ 0.2353 & 0.1701 & 0.9569 & 37.11 \end{array}\right)$$

**Fig 13:**
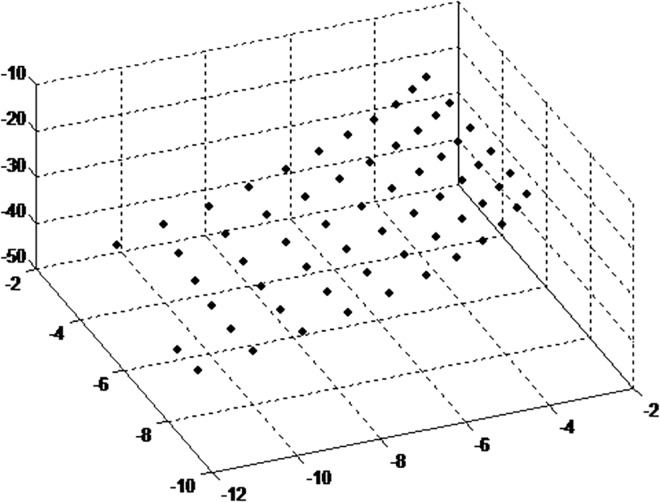
retrieval results based on proposed method.

**Fig 14:**
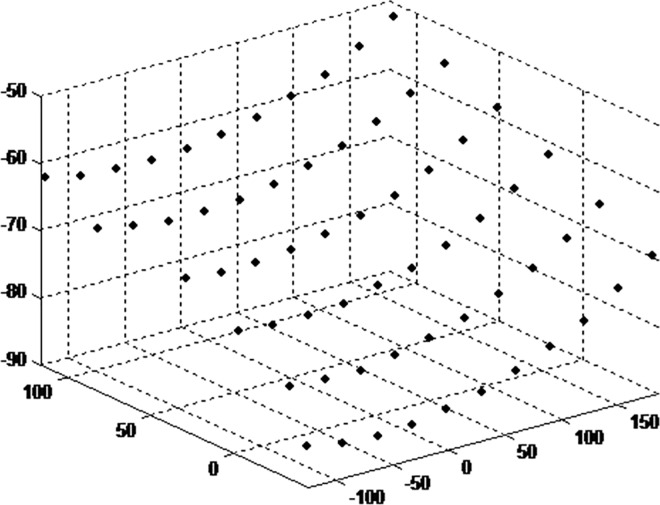
retrieval results based on Zhang’s method.

The diagrams of retrieval results are shown in Figs 13 and 14.

As shown in Figs 13 and 14, 3D point cloud are obtained successfully by both the methods. There is little difference in the results. The retrieval result of the plate is consistent with the result by Zhang’s method, thus the calibration result is close to Zhang’s method. The distances between adjacent 3D points are calculated. The distance results are gathered along horizontal and vertical direction.By Zhang’s method, the average distance in horizontal and vertical direction is 2.553, and the standard deviation is 0.01619. By the proposed method, the corresponding average distance and deviation is 2.648, 0.016724. It is shown that the proposed method can be used for multiple camera calibration conveniently.

## Conclusion

A novel study on camera calibration was carried out in the polar coordinate. There are several advantages of the proposed method. Some calibration image patterns are flexible in polar coordinate. Fewer calibration points are used. To calibrate multiple camera, the calibration images are placed according to the location of the camera. There is no accumulated error caused by transfer matrix. Zhang’s method, as well as the proposed method, are chosen for experimentation on both simulated and real data. The accuracy were evaluated. The efficiency of the proposed method is demonstrated in the experiments.

## Supporting Information

S1 File Data of intrinsic parameters. (XLSX)

S2 File Data of extrinsic parameters. (XLSX)

S3 File Data of parameters with 3 pictures. (XLSX)

S4 File Data of parameters with 10 pictures. (XLSX)
